# Reactivity and Penetration Performance Ni-Al and Cu-Ni-Al Mixtures as Shaped Charge Liner Materials

**DOI:** 10.3390/ma11112267

**Published:** 2018-11-13

**Authors:** Miao Sun, Chao Li, Xuguang Zhang, Xiaomin Hu, Xiaoyan Hu, Yingbin Liu

**Affiliations:** 1 School of Environment and Safety Engineering, North University of China, Taiyuan 030051, China; sunmiaoLS@163.com (M.S.); 18435132326@163.com (X.H.); huxy85@nuc.edu.cn (X.H.); 2 Beijing Special Vehicle Institute, Beijing 100000, China; cxlichao@163.com; 3 School of Mechanical Engineering, Nanjing University of Science and Technology, Nanjing 210094, China; zxg18801592268@163.com

**Keywords:** shaped charge liner, Ni-Al, energetic structural materials, penetration performance, microstructure analysis

## Abstract

Energetic structural materials (ESMs) have many potential military applications due to their unique functions. In this work, the reactivity and penetration performance of ESMs have been examined as a shaped charge liner material. The penetration experiments of nickel-aluminum (Ni-Al) and copper-nickel-aluminum (Cu-Ni-Al)-shaped charge liners (SCLs) have been designed and fired into 45# steel. The targets were recovered and analyzed by optical microscopy, electron microscopy, energy dispersive spectroscopy, and Vickers microhardness measurements. The head and tail of the crater walls penetrated by two reactive jets demonstrated unique microstructures. The jet rapidly decayed with the penetration process, but the “white” zone (a mixture of martensite and austenite) was more prominent in the tail, and the microhardness of the tail was much higher than that of the head. The results showed the continued exotherm of Ni-Al reactive jet when it was fired into the target. The addition of Cu reduced the exotherm of Ni-Al, Cu could not only increase the average crater size, but also raise the average penetration depth by 42%. These results offer valuable insight for utilizing ESM as shaped charge liner materials.

## 1. Introduction

Energetic structural materials (ESMs) or reactive metal materials (RMMs) possess desired mechanical properties and shock release energy. An ESM can be an ideal platform for the combined ballistic delivery of chemical and kinetic energies [1], and thus it can be utilized as reactive fragments and reactive liners for producing large and effective perforations in broad military applications. To achieve good properties of an ESM, the following three perspectives should be taken into account: (1) an ESM is sufficiently insensitive to the explosion environment during the acceleration of the explosion; (2) it has a sufficient penetration capability; and (3) it can combine kinetic energy with chemical reaction energy to increase its damage capacity [2,3]. Reactive metal materials have better density and sound velocity than fluorine-based reactive materials; thus, RMMs demonstrate better characteristics as shaped charge liner materials.

Nickel-aluminum (Ni-Al) powders are reactive metals that have been widely studied in recent years. A systematic review of the shock-induced chemical reaction (SICR) in Ni-Al powders can be found in the study from Thadhani [4]. Zhang [5] investigated the effect of sintering temperature on mechanical properties and energy density of Ni-Al materials. The results revealed that a certain amount of energy was transferred during the sintering procedure to enhance limited mechanical properties of Ni-Al materials. Subsequently, other metals were added to Ni-Al, and their SICR characteristics have shown an increase or decrease. Homan [6] studied the effects of the addition of three metals, namely magnesium (Mg), molybdenum (Mo), and copper (Cu), on the initiation and combustion properties of Ni-Al. The results showed that the addition of Cu significantly increased the initial SICR temperature and the reaction time. Xiong [7] analyzed the effects of Cu/PTFE on SICR behaviors and energy-releasing characteristics of Ni-Al composites. The experimental and theoretical results suggested that Cu could increase the critical shock pressure for the initiation of SICR and decreased the reaction efficiency under the same impact conditions.

The penetration performance of reactive-shaped charge liners (RSCL) has been widely investigated. Laszlo [8] studied RSCL fabricated by Zr_57_Nb_5_Cu_15.4_Ni_12.6_Al_10_. The results showed RSCL could form particle streams and the penetration depth of Zr_57_Nb_5_Cu_15.4_Ni_12.6_Al_10_ was shallower than that of the copper liner for the same structure (no chemical reaction), but it had better lateral damage. On the other hand, Ni-Al as a shaped charge liner material was analyzed in the stages of energy release. A triaxial loading system was designed by Philip [9] to determine shock reaction thresholds of Ni-Al reactive powders. The simple press tool could fragment Ni-Al RSCL, and the jet was recovered from the sand target. It also showed that Ni-Al RSCL reacted during the formation process of liner. However, it is challenging to determine whether the reaction would continue in the sand target due to the reaction exotherm.

Here we investigate whether Ni-Al reactive jets react in the steel target and how the penetration performance changes after passivation by addition of Cu. Two kinds of RSCLs made of Ni-Al and Cu-Ni-Al were studied for penetrating 45# steel targets. After the penetration experiments, the penetration depths and crater sizes of the RSCLs were compared. To investigate the reactivity of Ni-Al and the influence of Cu on SICR of Ni-Al when they penetrated the target as jets, we examined the microstructure of the crater walls. By comparing the microstructural differences between the head and tail of the crater walls, it could be inferred whether the jet released energy during the penetration progress. These investigations could provide a reference for the applications of ESM in shaped charge liners.

## 2. Materials and Methods

**Figure 1:**
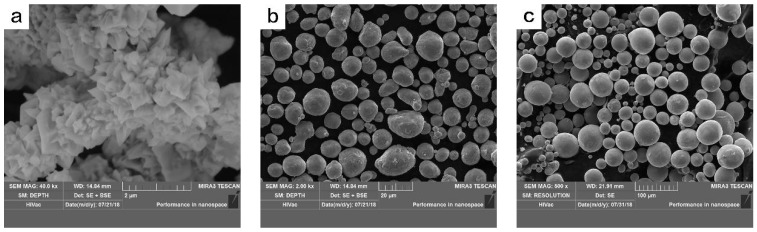
The powders used to prepare the reactive liners. (**a**) Ni; (**b**) Al; and (**c**) Cu.

The powders used to prepare the reactive liners were obtained by mechanical mixtures of Ni, Al, and Cu of different morphologies and sizes, as illustrated in Figure 1. The powders’ characteristics are listed in Table 1.

It is essential to produce RSCL without initiating any reactions to maximize the potential energy of RSCL. Powder metallurgy was implemented without sintering to produce all the RSCL. Table 2 shows the properties of two kinds of RSCL. Archimedes drainage method was used to measure the actual material density (AMD) of RSCL. The cross-sections of two kinds of RSCL were characterized by Mira3 LMH scanning electron microscope (SEM, Tescan, Brno, Czech Republic).

**Figure 2:**
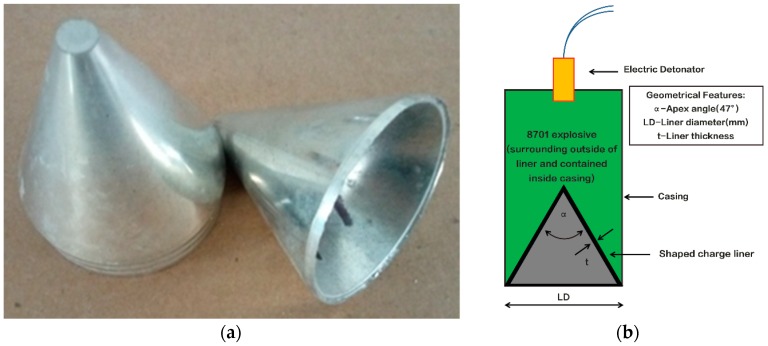
(**a**) Reactive-shaped charge liners and (**b**) the schematic of a shaped charge device.

**Figure 3:**
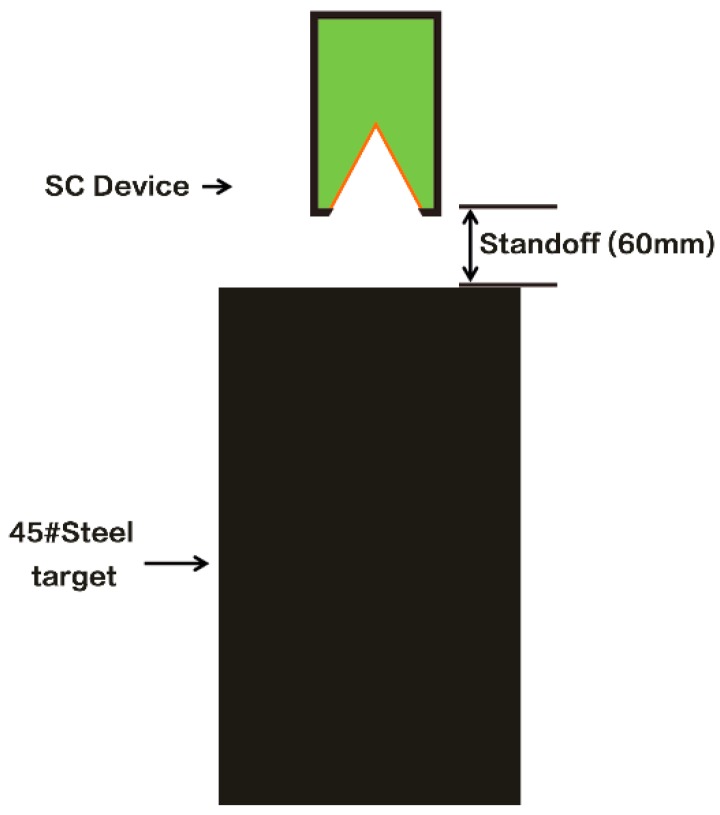
The schematic of the penetration experiments.

All liners used in these experiments had a conical geometry. The tolerance dimensions of the thickness (*t*) on the surface of the liners were less than 0.1 mm. Figure 2 shows RSCLs and the schematic of a shaped charge device; 45# steel was used as the shaped charge casing. The shaped charge was loaded with 8701, a hexogen-based (RDX) explosive. Charge diameter (*CD*) and the liner diameter (*LD*) were both 44 mm. The RSCLs were subjected to explosive loading under the same conditions, including stand-off distances, and 45# steel target columns as targets were used in the penetration experiments. The schematic of the penetration experiment is shown in Figure 3. The target columns were retrieved and cut along the centerline of the crater by electrical discharge machining. Two kinds of RSCLs were compared regarding their penetration performance. Some parts of the cross-section from the head and tail of the crater walls were cut from the targets, polished, and etched by the solution composed of 100 mL ethanol and 4 mL hydrogen nitrates to prepare samples for microstructural analysis. These samples were investigated by Axio Lab A1 optical microscope (OM, CarlZeiss, Jena, Germany), and SEM. X-MaxN Energy Dispersive Spectroscopy (EDS, Oxford-instruments, High Wycombe, UK) was applied along with SEM to determine the elements of the residual jet zone. To investigate the properties of different zones in penetration crater walls, we performed Vickers microhardness measurements (JMHVS-1000AT) using a standard diamond indenter at a 0.98 N load for 15 s.

## 3. Results

**Figure 4:**
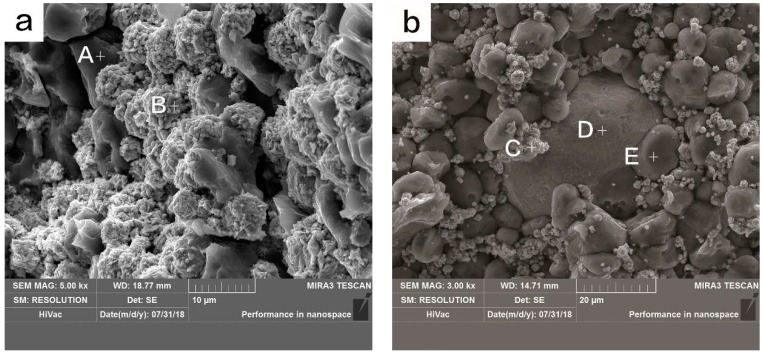
The SEM images of (**a**) Ni-Al RSCL and (**b**) Cu-Ni-Al RSCL.

**Figure 5:**
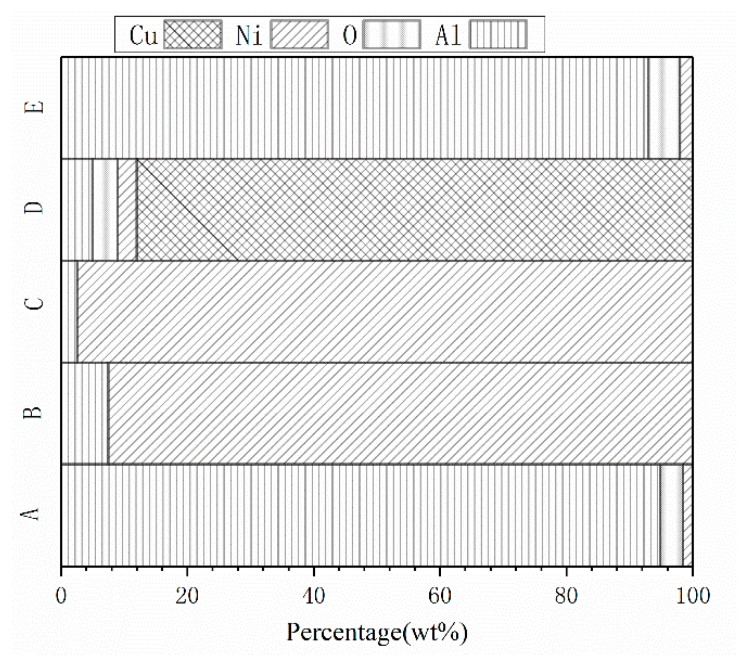
The point EDS results of A-E.

The SEM images of the fabricated Ni-Al and Cu-Ni-Al RSCLs are shown in Figure 4, and the EDS points results of A–E are shown in Figure 5. It indicates that A and E are Al powders, B and C are Ni powders, and D is Cu powder. Nickel particles tightly surrounded Al particles in Ni-Al RSCL, and Al particles had slight deformation. Figure 4b shows the microstructure of Cu-Ni-Al RSCLs, where larger Cu particles were embedded within the matrix of Ni and Al particles. 

**Figure 6:**
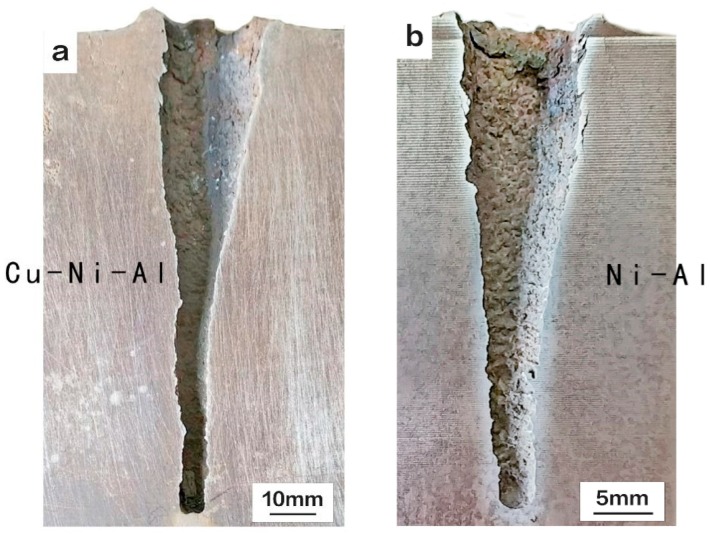
The macroscopic features of the targets penetrated by (**a**) Cu-Ni-Al and (**b**) Ni-Al jet.

Figure 6 shows the macroscopic targets penetrated by Ni-Al and Cu-Ni-Al reactive jets. The surface of the walls of crater were rough, and the crater sizes were relatively large. The entire penetration walls were clean, suggesting that no slug remained after penetration. The average penetration depth of Cu-Ni-Al jet was increased by 42% compared to the average penetration depth of Ni-Al jet. The data of penetration performance is shown in Table 3.

**Figure 7:**
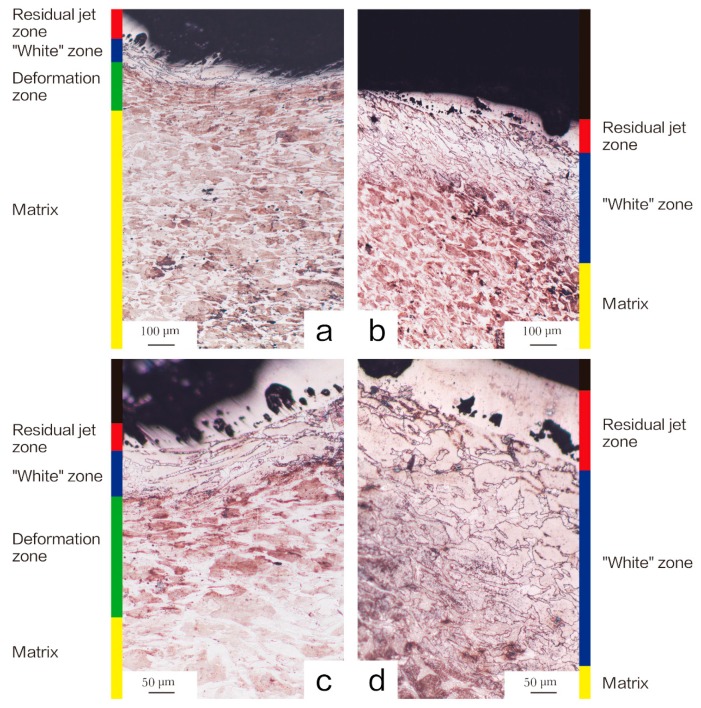
The microstructure of the crater walls penetrated by Ni-Al reactive shaped charge liner. (**a**) the head of the crater walls (100×); (**b**) the tail of the crater walls (100×); (**c**) the head of the crater walls (200×); (**d**) the tail of the crater walls (200×).

**Figure 8:**
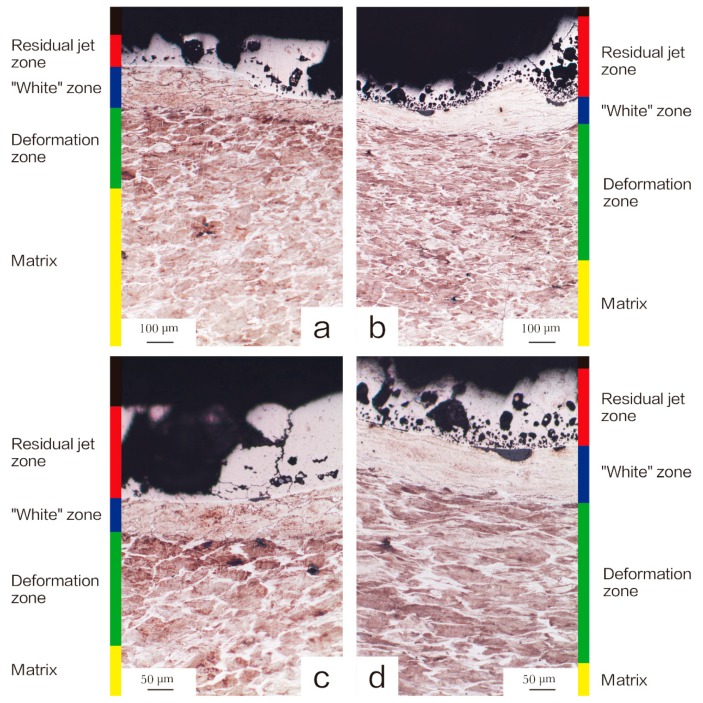
The microstructure of the crater walls penetrated by Cu-Ni-Al reactive-shaped charge liner. (**a**) the head of the crater walls (100×); (**b**) the tail of the crater walls (100×); (**c**) the head of the crater walls (200×); (**d**) the tail of the crater walls (200×).

Figure 7 and Figure 8 show OM images of the head and tail of the walls penetrated by Ni-Al and Cu-Ni-Al reactive jet. The microstructural characteristics indicate that the crater walls can be divided into four zones: the residual jet zone, “white” zone, deformation zone, and matrix. Table 4 lists the thickness parameters of the affected zones in crater walls. Residual jet zone and “white” zone in the head of the walls were thinner than that in the tail of the walls. No obvious deformation zone was observed in the tail of the walls penetrated by Ni-Al reactive jet. However, the deformation zone was observed in the tail of the walls penetrated by Cu-Ni-Al reactive jet.

Higher impact pressures tend to indicate higher temperature rises, and the head of the crater walls is subject to higher impact pressures from the jet, but it is worth noting that the “white” zone in the tail, encountering small impact pressure is much wider than that in the head. The severely deformed “white” zone and ferrite are visible from the head of the target, so the “white” zone of the head is mainly caused by jet impact. The “white” zone in the tail underwent severe deformation and received a large amount of released heat when the intermetallic compound was formed by Ni-Al. The exothermic heat of Ni-Al is enough to cause the self-propagating high-temperature synthesis (SHS) reaction, and the SICR also can be caused when shock wave passed through Ni-Al mixtures [10]. A critical question is whether these reactions are initiated only by the shock wave and proceed to their endings. After the shock wave passes through the material, the material may react further due to the combined action of shock and reaction exotherm. The wider “white” zone is absorbed by the tail due to more heat. Thus, the exothermic stage of the reactive jet can also occur at a later stage of penetration. Although at the microstructural level, the reaction is very complex and depends on a number of factors such as the porosity of material and size and shape of particles, at this condition of the powders, Ni-Al jet has not only a specific penetrating ability but also the ability to radiate heat inside the target.

**Figure 9:**
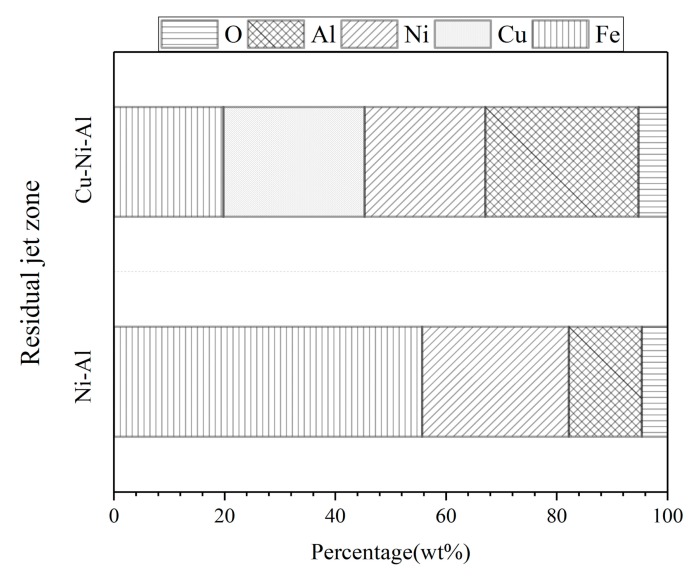
The point EDS results of Ni-Al and Cu-Ni-Al residual jet zone.

**Figure 10:**
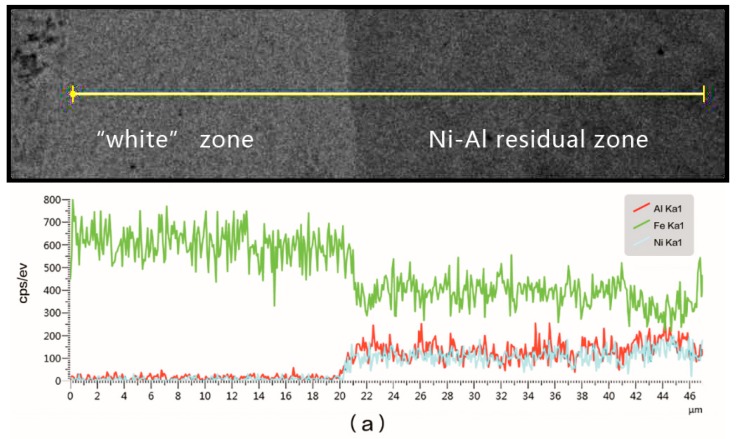

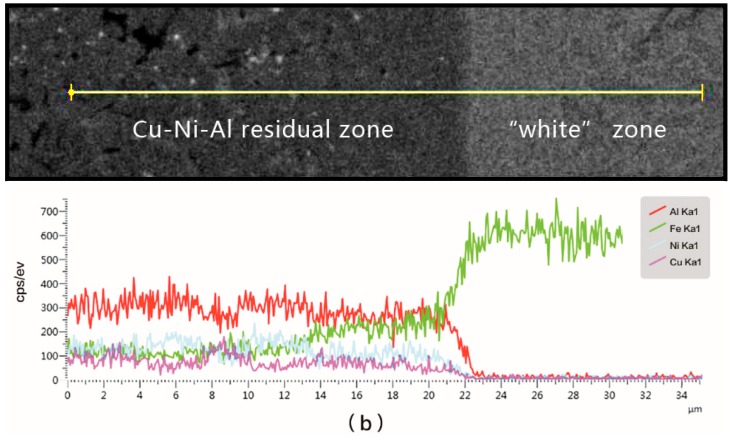
The EDS line analysis at the interface between (**a**) Ni-Al residual zone and “white” zone and (**b**) Cu-Ni-Al residual zone and “white” zone.

The point EDS results in the residual zone are shown in Figure 9, while Figure 10 shows the EDS line analysis results between the residual and “white” zone. The percentage of Fe in Ni-Al residual jet was much higher than that in Cu-Ni-Al residual jet. The percentage of oxygen was low in both of the residual jets. Nickel and Al signal intensities in Ni-Al residual jets were similar and stable, while the strength transition of Fe signal was rapid, demonstrating a large shift in the magnitude. The signal intensities of Ni and Al in Cu-Ni-Al residual jets were quite different, and the transition of Fe signal intensity was gentle, experiencing a very weak change in intensity.

**Figure 11:**
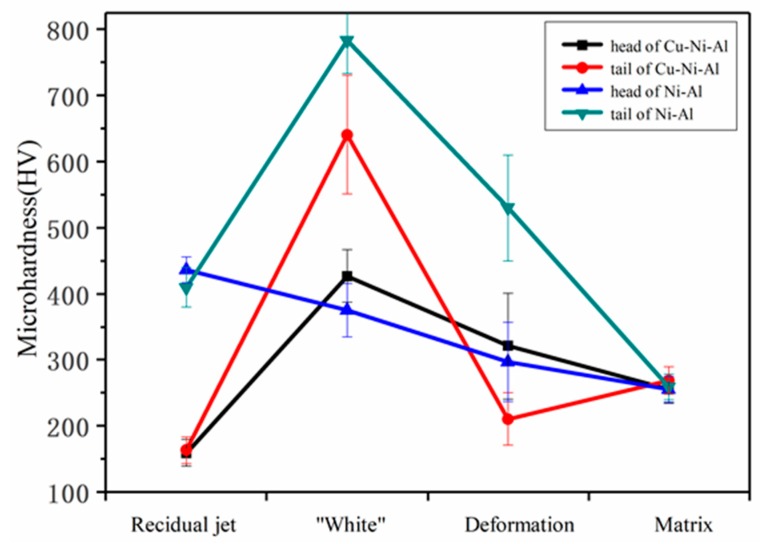
The Vickers hardness values for all zones.

The Vickers microhardness results are shown in Figure 11. In the homogeneous residual jet zone, the microhardness values in the head were similar to that in the tail. The microhardness values of Ni-Al residual jet zone were doubled than that of Cu-Ni-Al. In the homogeneous “white” zone of target penetrated by two RSCL, the microhardness values in the tail were greater than that in the head. The microhardness values of Ni-Al “white” zone in the tail were increased 150 HV than that of Cu-Ni-Al. 

## 4. Discussion

The particle size matching principle is helpful to reduce the porosity of the powder material, although the optimal porosity for the inert jet (Cu or W-Cu) is advantageous to improve the cohesiveness of the liner forming the jet, thereby improving the penetration performance of the liners [11]. However, when the porosity is too large, high-energy storing capacity could easily induce the shock-induced chemical reaction of the reactive powder material; thus, high porosity may cause irreversible bursting of jets [12] and reduce their ability to penetrate the target. Therefore, compared with the porosity of Ni-Al and Cu-Ni-Al RSCLs, the addition of large Cu particles can effectively reduce the porosity of the liners, thereby enhancing the penetrating performance of the reactive jets.

The comparison of the penetrating properties suggests that, Cu provided an apparent increase in the average crater size and penetration depth of Ni-Al reactive liner. From a conventional point of view, the depth of penetration is dominated by the density of the liner and target, $P=L(\frac{\rho_{j}}{\rho_{t}}{)}^{\frac{1}{2}}$, where $\rho_{j}$ is the density of the jet, $\rho_{t}$ is the density of the target, and L is the length of the jet that is mostly decided by stand-off when the stand-off is very short. The densities of two reactive liners and target materials were the same, and the same stand-off was used in the penetration experiments. Thus, it can be inferred that Cu enhanced the penetration properties of Ni-Al reactive liners. Compared with the previous W and W-Cu jet penetration experiments [13], the addition of Cu effectively reduced the interaction between the jet and target, decreased the formation of a hard phase of the target, and thus improve the penetration depth. The crater size is also an important indicator of penetration performance since it is the reflection of the total energy of the jet. Although Cu increased the penetration depth of Ni-Al reactive jet, it had no adverse effect on the crater size; thus, Cu could serve as a tool to engineer the properties of Ni-Al ESM for the applications in shaped charge materials.

By comparing the microscopic images of the head and tail of the ballistics, the speed of the jet rapidly decreased with the penetration depth. As shown in Figure 3, the jet adhered to the surface of crater walls finally due to the low speed, so the residual jet width of the tail was higher than that of the head. It is known that the deformation process at high strain rates is adiabatic, and for most metals, 90% of the deformation is converted into heat. Steel undergoes a phase transition from α (Body-Centered Cubic) to γ (Faced-Centered Cubic) at 912 °C [14]. If the temperature within the crater walls reaches this value, then the phase transformation occurs, and this phase can be retained at room temperature, since the material within the crater walls is much smaller than the entire target, and is rapidly quenched by the surrounding material once plastic deformation ceases. Therefore, it is reasonable that the “white” zone, which contacts with residual jet have undergone phase transformation. The martensitic transformation is generally unlike for the surrounding material. The EDS analysis results and the hardness measurements confirm the martensitic transformation took place. The EDS results shown that Ni, Al, and Cu in the residual jet maintained their initial percentage. The percentage of Fe in the residual jet indicates the intensity of the interaction with the target [15]. Cu reduces the interaction between the jet and target and decreases the degree of Ni-Al reaction that is evident from the signal strengths of the elements. This outcome is in agreement with other studies by Homan [5] and Xiong [7].

The hardness values of the zones in the target can explain the microstructural phenomenon better. Yin [16] proved that due to the extremely high temperature on the crater walls and the high cooling rate, the “white” zone comprises a mixture of martensite and retained austenite. This microstructure is the reason why the “white” zone is not as hard as untampered martensite of steel. In this study, because of the further reaction of Ni-Al, the residual austenite in the “white” zone of the tail was transformed into martensite. The hardness of the “white” zone was greatly enhanced due to the increase of the martensitic phase. This result indirectly proves that the Ni-Al reaction occurred after the penetration was complete. The hardness of the Cu-Ni-Al residual jet zone was much lower than that of Ni-Al and even smaller than the hardness of the matrix. In other words, Ni-Al jet forms intermetallic compounds that increase the hardness, while Cu weakens the reaction between Ni-Al and reduces the number of intermetallic compounds.

## 5. Conclusions

The penetration experiments were performed on two kinds of shaped charge liners, Ni-Al and Cu-Ni-Al. The average penetration depth of Cu-Ni-Al jet was increased by 42% compared to the average penetration depth of Ni-Al jet. The microstructures of the targets were analyzed to investigate Ni-Al reactive jet on SICR behaviors and the effect of Cu on SICR behaviors of Ni-Al jet. Although many issues, including the mesoscale characteristics of powders or the structures of shaped charge could affect the SICR behaviors of Ni-Al jet, our results demonstrate that Ni-Al reactive jet did not completely react after the shock wave, and could release heat energy after penetration. Little oxidation occurred at 60 mm standoff in Ni-Al and Cu-Ni-Al reactive jet, and the energy that caused the microstructural change of the targets was mainly provided by plastic deformation and exothermicity when the intermetallic compounds were formed. The results of the penetration experiments, EDS, and Vickers microhardness measurements indicate that the addition of Cu not only reduces the initial SICR behavior of the Ni-Al jet but also enhances the penetration performance and subsequent release behavior of Ni-Al reactive jet. The purpose of using RSM in shaped charge liners is to keep little or no reaction while the explosive is driving the liners, have specific penetration capability, and have enough reactivity when the jet penetrates the target and then releases more energy to the target. Based on our analysis, Cu enables Ni-Al to have these characteristics that could provide new insight into novel warhead design.

Changing standoff distance may increase the length of the jet or break up the jet since the penetration performance would vary widely. The reaction of Ni-Al and the interaction between the jet and target will not be similar based on the different mass fraction of Cu. In future work, we aim to vary the standoff and mass fraction of Cu for the reactive jet to understand the influence of standoff and mass fraction of Cu on RSCL penetration performance. 

## Figures and Tables

**Table 1:** The characteristics of powder mixtures.

Material	Shape	Size (μm)	Purity (Pct)
Nickel Powder	thorny rounded	2–6	99.9
Aluminum Powder	rounded	6–15	99.9
Copper Powder	rounded	20–50	99.9

**Table 2:** The properties of two kinds of reactive-shaped charge liners (RSCLs).

Number	Type	wt (%)	Mass (g)	AMD (g/cm^3^)	Height (mm)	Thickness (mm)	Porosity (%)
A-1	Ni-Al	66:34	27.5	4.73	47	1.78	6.2
A-2	Ni-Al	66:34	27.9	4.76	47	1.78	5.5
A-3	Ni-Al	66:34	27.4	4.75	47	1.78	5.7
B-1	Cu-Ni-Al	30:35:35	27.3	4.78	47	1.72	3.3
B-2	Cu-Ni-Al	30:35:35	27.7	4.75	47	1.78	3.9
B-3	Cu-Ni-Al	30:35:35	27.5	4.77	47	1.75	3.5

**Table 3:** The penetration depth and entrance aperture data of RSCLs.

Number	Penetration Depth (mm)	Average Penetration Depth (mm)	Crater Size (mm × mm)	Average Crater Size (mm × mm)
A-1	75	76	20 × 20	21 × 20
A-2	78		21 × 20	
A-3	76		21 × 20	
B-1	105	108	23 × 25	22 × 23
B-2	112		21 × 22	
B-3	109		23 × 22	

**Table 4:** The thickness parameters of the affected zones penetrated by two kinds of RSCLs.

SCL Materials	Part of Crater Wall	Residual Jet Zone (μm)	“White” Zone (μm)	Deformation Zone (μm)	Total Affected Zone (μm)
Ni-Al	head	10–90	40–80	120–170	170–240
Ni-Al	tail	30–130	230–300	-	250–300
Cu-Ni-Al	head	50–220	80–150	200–270	330–410
Cu-Ni-Al	tail	80–350	100–200	200–300	380–500
